# Indications for and Outcomes of Three Unilateral Biportal Endoscopic Approaches for the Decompression of Degenerative Lumbar Spinal Stenosis: A Systematic Review

**DOI:** 10.3390/diagnostics13061092

**Published:** 2023-03-14

**Authors:** Anh Tuan Bui, Giam Minh Trinh, Meng-Huang Wu, Tung Thanh Hoang, Ming-Hsiao Hu, Jwo-Luen Pao

**Affiliations:** 1 International Ph.D. Program in Medicine, College of Medicine, Taipei Medical University, Taipei 110, Taiwan; 2 Department of Spine Surgery, Military Hospital 103, Vietnam Military Medical University, Hanoi 10000, Vietnam; 3 Department of Trauma-Orthopedics, College of Medicine, Pham Ngoc Thach Medical University, Ho Chi Minh City 70000, Vietnam; 4 Hospital for Traumatology and Orthopedics, Ho Chi Minh City 70000, Vietnam; 5 Department of Orthopedics, School of Medicine, College of Medicine, Taipei Medical University, Taipei 110, Taiwan; 6 Department of Orthopedics, Taipei Medical University Hospital, Taipei 110, Taiwan; 7 Department of Orthopedic Surgery, National Taiwan University Hospital, Taipei 100, Taiwan; 8 Department of Orthopedic Surgery, Far Eastern Memorial Hospital, New Taipei City 220, Taiwan; 9 General Education Center, Lunghwa University of Science & Technology, Taoyuan 333, Taiwan

**Keywords:** endoscopy, biportal, spinal stenosis, lumbar vertebrae

## Abstract

Objective: In this systematic review, we summarized the indications for and outcomes of three main unilateral biportal endoscopic (UBE) approaches for the decompression of degenerative lumbar spinal stenosis (DLSS). Methods: A comprehensive search of the literature was performed using Ovid Embase, PubMed, Web of Science, and Ovid’s Cochrane Library. The following information was collected: surgical data; patients’ scores on the Visual Analog Scale (VAS), Oswestry Disability Index (ODI), and Macnab criteria; and surgical complications. Results: In total, 23 articles comprising 7 retrospective comparative studies, 2 prospective comparative studies, 12 retrospectives case series, and 2 randomized controlled trials were selected for quantitative analysis. The interlaminar approach for central and bilateral lateral recess stenoses, contralateral approach for isolated lateral recess stenosis, and paraspinal approach for foraminal stenosis were used in 16, 2, and 4 studies, respectively. In one study, both interlaminar and contralateral approaches were used. L4-5 was the most common level decompressed using the interlaminar and contralateral approaches, whereas L5-S1 was the most common level decompressed using the paraspinal approach. All three approaches provided favorable clinical outcomes at the final follow-up, with considerable improvements in patients’ VAS scores for leg pain (63.6–73.5%) and ODI scores (67.2–71%). The overall complication rate was <6%. Conclusions: The three approaches of UBE surgery are effective and safe for the decompression of various types of DLSS. In the future, long-term prospective studies and randomized control trials are warranted to explore this new technique further and to compare it with conventional surgical techniques.

## 1. Introduction

Degenerative lumbar spinal stenosis (DLSS) is a common disease with a prevalence of 20–25% in the general population, and the prevalence tends to increase in individuals aged >60 years [1]. Patients with DLSS present with various symptoms, such as low back pain, radicular leg pain, neurologic deficit, and intermittent claudication, which negatively affect their quality of life.

In the case of the failure of conservative treatments for moderate to severe DLSS, surgery is the optimal alternative. A conventional surgical procedure involves a large incision, extensive soft tissue dissection, and wide laminectomies with or without concomitant spinal fusion [2]. Recently, minimally invasive surgery has been demonstrated to have surgical outcomes compatible with those of conventional surgery. Minimally invasive surgery has several advantages, such as a small incision, minimal soft tissue injury, and preservation of the stabilizing structures, which helps preserve the physiological function of the lumbar spine [3]. Unilateral biportal endoscopic (UBE) surgery is a minimally invasive surgical technique. This technique has undergone rapid developments in the last two decades. UBE surgery is performed through two percutaneous portals: one facilitates endoscope insertion and saline inflow, whereas the other serves as the working portal and facilitates saline outflow. Because of hydrostatic pressure and continuous normal saline flow, the endoscopic field is almost bloodless, bright, clear, and magnified. The working portal enables surgeons to freely use various surgical instruments. Because the endoscope and the surgical instruments can be maneuvered separately with no limitation from any tubular retractors, surgeons can operate efficiently and ergonomically.

**Figure 1:**
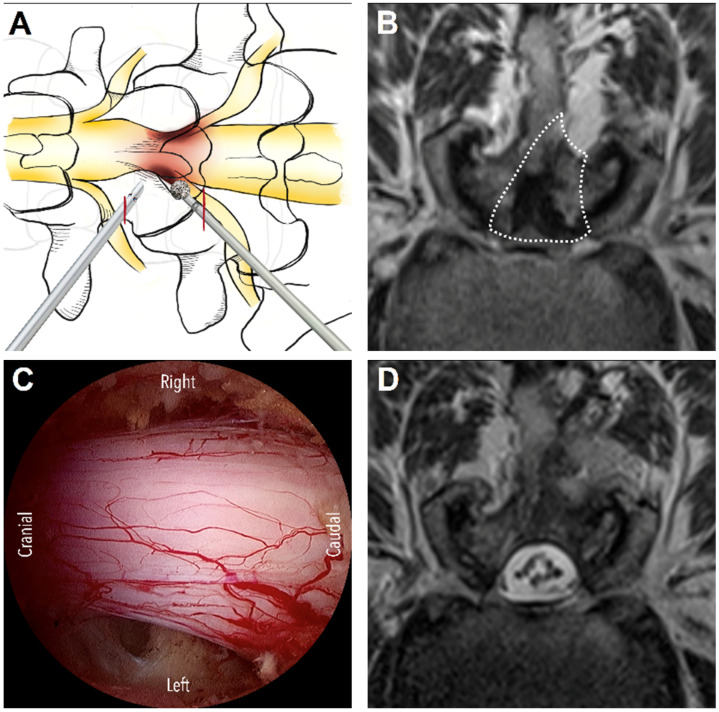
The interlaminar approach. (**A**) The schematic diagram illustrates the skin incisions (red lines) and initial target area of decompression from the left side: the junction of spinous process and lamina. (**B**) Pre-operative planning for bilateral decompression via unilateral laminotomy on MRI (dashed line area). (**C**) The endoscopic view demonstrates the complete decompression of central and bilateral lateral recess stenosis. (**D**) Postoperative MRI shows good decompression with good preservation of the facet joints and minimal soft tissue damage.

**Figure 2:**
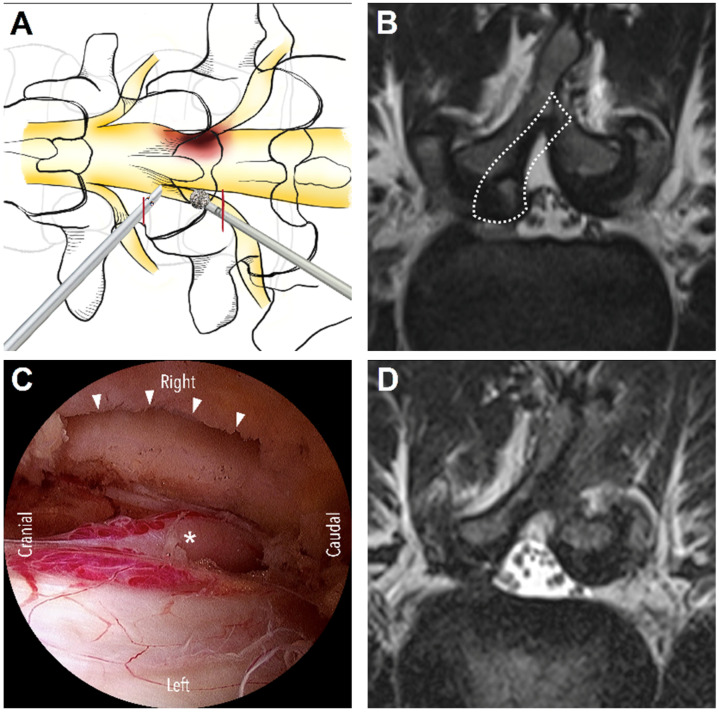
The contralateral approach. (**A**) The schematic diagram illustrates the skin incisions (red lines) and initial target area of decompression from the left side: the junction of spinous process and lamina. (**B**) Pre-operative MRI shows isolated lateral recess stenosis and contralateral decompression via an opposite interlaminar window (dashed line area). (**C**) The endoscopic view demonstrates the facet joint viewed from inside the spinal canal (white arrow heads), decompression of the lateral recesses, and the traversing nerve root (white asterisk). (**D**) Post-operative MRI shows adequate decompression of the lateral recess with complete preservation of the ipsilateral facet joints and minimal soft tissue damage.

**Figure 3:**
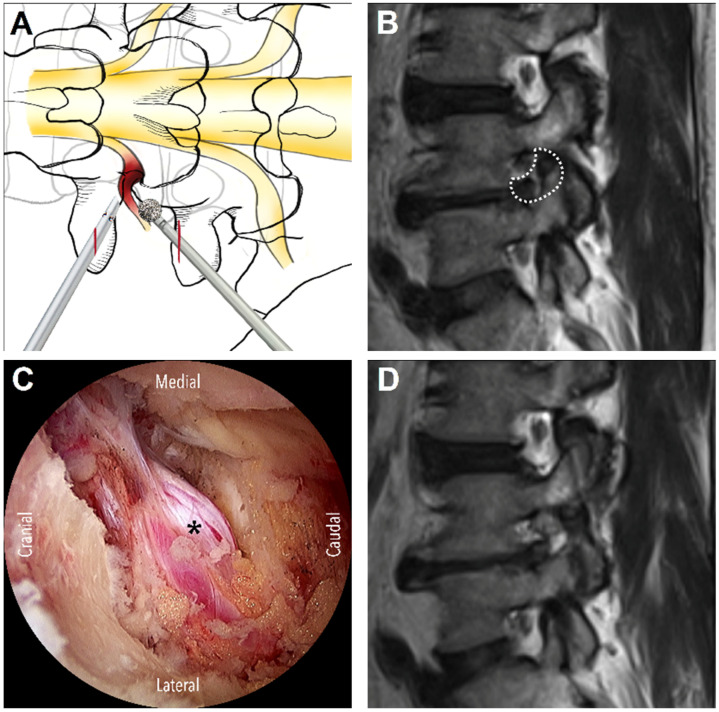
The paraspinal approach. (**A**) The schematic diagram illustrates the skin incisions (red lines) and initial target area of decompression from the left side: the isthmus of pars interarticularis or the tip of superior articular process. (**B**) Pre-operative MRI shows the foraminal stenosis, entrapment of the nerve root, and planned foraminal decompression (dashed line area). (**C**) The endoscopic view demonstrates decompression of the foramen and the exiting nerve root (asterisk). (**D**) Post-operative MRI shows widening of the foraminal space for the nerve root.

On the basis of anatomical considerations, DLSS can be classified as central, lateral recess, or foraminal stenosis [4]. The suitable surgical approaches vary across stenosis types. Three main approaches are used for UBE surgery: interlaminar, contralateral, and paraspinal. The interlaminar approach is the most commonly used approach in UBE decompression and is generally indicated for central stenosis and lateral recess stenosis. For this approach, the unilateral laminotomy for bilateral decompression (ULBD) technique, which was introduced by Guiot et al. [5], is generally used. First, ipsilateral laminotomy is performed, followed by sublaminar decompression to decompress the contralateral lateral recess and then ipsilateral medial facetectomy to decompress the ipsilateral lateral recess. After removing the hypertrophic ligamentum flavum, the central canal, bilateral lateral recesses, and bilateral traversing nerve roots may be decompressed effectively (Figure 1). For patients with unilateral radiculopathy due to isolated lateral recess stenosis, the contralateral approach is a favorable alternative. In this approach, first, sublaminar decompression is performed through the interlaminar window, followed by the removal of the ligamentum flavum and then the decompression of the contralateral lateral recess. The lateral recess on the symptomatic side can be effectively decompressed with minimal injury of the facet joints [6]. The ipsilateral facet joint is completely preserved (Figure 2). The paraspinal approach is used for foraminal or extraforaminal stenosis (Figure 3). This approach is similar to the Wiltse approach [7], which is used in conventional open surgeries; however, an endoscope is used in the paraspinal approach to avoid excessive dissection of the paraspinal muscles. The paraspinal approach is an ideal option to access the foraminal area at the L5-S1 level, even if a patient has a high iliac crest [8]. Although several studies have explored the UBE technique and its outcomes in patients with DLSS, to the best of our knowledge, no study has focused on systematizing the different approaches commonly used for UBE surgery. Therefore, in the present study, we reviewed the relevant literature to systematically report the surgical outcomes of the three main UBE approaches for DLSS decompression.

## 2. Materials and Methods

### 2.1. Search Strategy and Selection Criteria

The PRISMA (Preferred Reporting Items for Systematic Reviews and Meta-analysis) [9] statement was used for this systematic review (Supplementary Material File S1). We searched several databases, including Ovid Embase, PubMed, Web of Science, and Ovid’s Cochrane Library, for articles published between 1950 and (15 March) 2020. On the basis of the patients/population, intervention, comparator, and outcomes approach, the search strategy was designed as follows: patients with DLSS (patients/population) undergoing UBE surgery (intervention) performed using one of its three main approaches, namely interlaminar, contralateral, and paraspinal (comparator) and relevant surgical outcomes. The search string (containing keywords or MeSH terms) was as follows: (“UBE” OR ((“biportal” OR “two portal” OR “dual portal”) AND (“endoscopic”))) AND (“lumbar”).

We included articles describing UBE surgery performed for a population of more than 10 patients with DLSS. An additional inclusion criterion was the availability of information on demographics, surgical approach, and clinical outcomes. The following articles were excluded: articles on lumbar disk herniation, spinal fusion, or cervical or thoracic spines; articles not written in English; and case reports, technical reports, animal studies, review articles, or meta-analyses. For studies conducted by the same author groups using an overlapping patient population and similar research methods, we included only the relatively recent study with comparatively extensive clinical data.

### 2.2. Data Extraction and Quality Assessment

The title and abstract of each article obtained from our search results were evaluated by two independent reviewers (A.T.B. and G.M.T.) based on the aforementioned criteria. A list of potential articles was prepared for manual review. Articles were selected for full-text review if they met the study inclusion criteria as per the agreement between the reviewers, with a low threshold for retrieval. In addition, the reference list of each included article was searched manually to include further relevant articles. Any inconsistencies between the two authors were resolved through a discussion with another author.

The following data were extracted: general characteristics of the study, number of patients and involved spinal levels, design of the study, age and sex of patients, duration of follow-up, indication for surgery, and clinical outcomes after surgery. We primarily collected general data to determine the differences among the three main UBE approaches. To evaluate postoperative clinical outcomes, we collected data on patients’ scores on the Visual Analog Scale (VAS; for back and leg pain) [10], Oswestry Disability Index (ODI) [11], and Macnab criteria [12] (the proportion of excellent and good results). Two authors (A.T.B. and G.M.T.) were responsible for data retrieval—one extracted data, and the other evaluated data accuracy. All data were summarized qualitatively and through simple synthesis. There was no statistical collecting or performing of a meta-analysis.

For the quality assessment, randomized controlled trials (RCTs) were evaluated using version 2 of the Cochrane risk-of-bias tool for randomized trials (RoB 2) by two reviewers (A.T.B. and G.M.T.) [13]. Other non-RCTs studies were assessed using the tool adopted from the National Institutes of Health/National Heart, Lung and Blood Institute (NIH) for case control studies and case series studies (available at: http://www.nhlbi.nih.gov/health-topics/study-quality-assessment-tools, accessed on 8 February 2023). Any discrepancies that arose throughout this procedure were discussed with a third author (J.L.B.).

## 3. Results

### 3.1. Search Results

**Figure 4:**
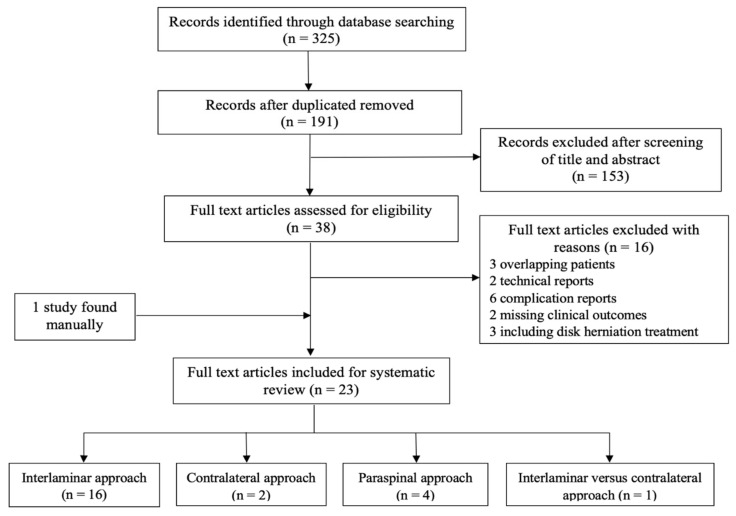
PRISMA flow chart for study selection.

Figure 4 depicts the search process and results. Initially, we identified a total of 325 articles from the aforementioned four databases. After removing duplicate and irrelevant articles through screening the abstracts, we selected a total of 38 articles for full-text review. After careful inspection, we further excluded 16 articles based on our article inclusion criteria (three studies included overlapping patient populations, two were technical reports, six were complication reports, two reported no clinical outcomes, and three did not focus on DLSS; Figure 4). One study was included through manual search [14]. Finally, we reviewed a total of 23 studies comprising 7 retrospective comparative studies, 2 prospective comparative studies, 12 retrospectives case series, and 2 randomized controlled trials.

### 3.2. Study Characteristics

For two RCTs studies, one was judged to have low risk of bias, and one was judged to have some concern of bias. All other studies were judged as “good” or “fair quality” by NIH quality assessment tool. The summary and description of the risk of bias assessment is presented in Supplementary Material File S2.

Among the 23 included studies, 16, 2, and 4 reported on the interlaminar, contralateral, and paraspinal approaches, respectively; one study focused on both the interlaminar and contralateral approaches. Table 1, Table 2 and Table 3 summarize the characteristics of the included studies stratified on the basis of the three main UBE approaches. However, a study conducted by Yeung et al., which focused on the comparison of the outcomes of interlaminar UBE decompression and contralateral UBE decompression, is not included in the aforementioned three tables. This study focused on the treatment of DLSS with predominant unilateral lower limb neurogenic claudication or neurological symptoms. In the aforementioned retrospective study, the interlaminar approach was used for 37 patients (mean age, 66.6 ± 13.6 years), whereas the contralateral approach was used for 34 patients (mean age, 65.8 ± 12.3 years) [15].

According to our review, the interlaminar approach was used for a total of 884 patients, whereas the contralateral and paraspinal approaches were used for a total of 74 and 103 patients, respectively (Table 4). The mean age of patients was as follows: interlaminar UBE approach group, 64.11 years (range, 52–71.2 years); contralateral UBE approach group, 61.37 years (range, 57.3–65.8 years); and paraspinal approach group, 65.65 years (range, 59.5–70.5 years). L4-5 was the most common level operated using the interlaminar and contralateral approaches, accounting for approximately 45% of the total cases. In contrast, L5-S1 was the most common level operated using the paraspinal approach, accounting for approximately 56.88% of the total cases.

The interlaminar approach was used primarily for patients with central stenosis or bilateral neurological symptoms. In a total of three studies reporting on the contralateral approach, the included patients typically presented with unilateral radicular symptoms. The paraspinal approach was consistently indicated for patients with foraminal or extraforaminal stenosis.

### 3.3. Surgical and Clinical Outcomes

Table 5 summarizes the surgical and clinical outcomes. Among the three approaches, the mean operation time of the interlaminar approach was the shortest at 64.24 min (range, 36–98.3 min; 15 articles). The operation time was 77.27 min (range, 60.1–102.5 min) for the contralateral approach (3 articles) and 70.43 min (range, 48.7–96.7 min) for the paraspinal approach (4 articles). The longest operation time (102.5 ± 43.66 min) was reported by Akbary et al. [6] for the contralateral approach. They included patients with unilateral radiculopathy due to spinal stenosis at two levels (lateral recess and foraminal (at the cranial adjacent level) stenoses). All patients in their study had undergone surgery through the contralateral approach, but two nerve roots had to be decompressed in these patients. Thus, the operation time was prolonged.

Patients’ VAS and ODI scores were calculated as follows: (preoperative value − final follow-up value)/preoperative value × 100%. The recovery rate for patients with leg pain who had undergone surgery following the interlaminar approach (73.46%) was higher than that for those who had undergone surgery following the contralateral and paraspinal approaches (63.6% and 70.9%, respectively). Improvements in patients’ VAS scores for back pain were satisfactory after interlaminar and contralateral UBE surgeries (69.96% and 78.66%, respectively), but the improvement was only 53.65% after paraspinal UBE surgery. Improvements in patients’ ODI scores were similar among the three approaches (approximately 70%). Considering that a total of 10 studies examined the interlaminar approach, the ratio of good and excellent outcomes according to the Macnab criteria was 87.22% (range, 76.66–94.4%). This ratio was 78.23% for studies on the paraspinal approach. None of the studies focusing on the contralateral approach reported Macnab outcomes.

For the interlaminar and contralateral approaches, the most common complication was dural injury, followed by postoperative epidural hematoma (Table 4). The overall complication rates were 5.7%, 4.05%, and 1.94% for the interlaminar, contralateral, and paraspinal approaches, respectively (Table 5).

## 4. Discussion

The UBE technique has been widely used for treating DLSS [21,36,37]. The aforementioned three major UBE approaches have been widely used in the treatment of most degenerative lumbar spine pathologies, which are conventionally treated using open or microscopic surgery. The findings of the present study revealed that all three UBE approaches are safe and effective. However, the indications and rationales for the approaches vary across pathologies.

The interlaminar approach is the most frequently used for the decompression of DLSS. Based on our search results, this approach was used in 17 studies, whereas the contralateral and paraspinal approaches were used in 3 and 4 studies, respectively. In the interlaminar approach, the central canal, ipsilateral lateral recess, and contralateral lateral recess are decompressed through a small laminotomy of the ipsilateral lamina following the concept of ULBD [14,28,30]. This decompression technique is advantageous for patients presenting with bilateral lower limb symptoms or neurogenic claudication due to moderate to severe canal stenosis, which is the most frequent clinical presentation of patients with DLSS; this explains the wide use of the interlaminar approach. The articles reported considerable reduction in patients’ VAS scores for low back and leg pain; of the patients, 87% exhibited good to excellent Macnab outcomes. However, most studies reported short-term outcomes; the longest follow-up duration was 28 months [14].

To reduce the possibility of post-decompression segmental instability, the integrity of the facet joints must be preserved to the highest possible extent [38]. Although the resection of the medial aspect of the facet joint is inevitable for the adequate decompression of the lateral recess, the UBE technique provides excellent preservation of the facet joints [17,20,25]. Although we found only two studies with no evidence of segmental instability after UBE decompression, the follow-up periods were too short to draw robust conclusions [21,22].

According to our review results, the preservation of the facet joint was better on the contralateral side than on the ipsilateral side when decompression was performed through unilateral laminotomy [17,20,25]. Therefore, for patients with isolated lateral recess stenosis who present with unilateral radicular symptoms, the contralateral approach may be favorable for ensuring the adequate decompression of the nerve root while preserving the contralateral facet joint. In the studies we reviewed, the ipsilateral facet joint was 100% preserved. Most of the ligamentum flavum and epidural fat can be preserved to prevent epidural adhesion. Juxtafacet or intraspinal facet cyst is a distinct cause of isolated lateral recess stenosis. The cyst may be exposed and removed through the contralateral approach, with minimal disturbance to the facet joint [31]. Moreover, in patients with severe degeneration and exaggerated hypertrophy of the facet joint and spinous process, the interlaminar space on the side with relatively severe symptoms may become too small to advance the endoscope and surgical instruments. The contralateral approach may be a favorable alternative.

The contralateral approach is advantageous for patients with the compression of two adjacent nerve roots in the lateral recess. After sublaminar decompression, the endoscope and surgical instruments can be advanced deep into the contralateral lateral recess to decompress the exiting root of the cranial vertebra, neural foramen, and traversing nerve root of the caudal vertebra through a single contralateral approach [6]. We only found three studies on the contralateral approach [6,15,31]; nonetheless, some studies included patients with unilateral radiculopathy. The contralateral approach is more technically demanding than the interlaminar approach; furthermore, the contralateral approach is associated with a potentially higher risk of dural tears (when gaining access to the contralateral side). This may be the reason why most surgeons prefer the interlaminar approach to directly treat ipsilateral pathologies.

Total facetectomy with/without fusion is considered the standard surgical treatment for lumbar foraminal stenosis [39]. Total facetectomy is associated with the risk of segmental instability, and fusion may lead to a variety of complications, such as adjacent segment degeneration, pseudoarthrosis, implant failure, and chronic low back pain due to the atrophy or fibrosis of back muscles [40,41]. Facet joint-preserving foraminal decompression through the Wiltse approach was first performed using microscopic or microendoscopic techniques. However, the outcomes were unfavorable (e.g., incomplete decompression and segmental instability due to excessive bony resection), which necessitated subsequent fusion surgery [42,43,44]. Of the foraminal or extraforaminal stenosis cases, 75% occurred at L5-S1 with the entrapment of the L5 exiting nerve root [39]. This foramen was confined by the hypertrophic facet joint, sacral ala and iliac crest, iliolumbar ligament, and L5 transverse process. These anatomical barriers and the high bleeding tendency from muscle dissection render the L5-S1 foramen extremely difficult to access using the microscopic or microendoscopic technique [45]. Through the UBE technique, bleeding may be suppressed using normal saline; moreover, the endoscope and surgical instruments may be advanced extremely close to the foramina and nerve roots to overcome anatomical barriers. Thus, adequate foraminal decompression with the preservation of the facet joint can be ensured even at L5-S1. Among the surgeries involving the paraspinal approach, 56.9% were performed for L5-S1 foraminal or extraforaminal decompression. All four studies on the paraspinal approach reported considerable improvement in patients’ VAS scores for leg pain and ODI scores without any post-decompression segmental instability or the requirement for subsequent fusion. However, the longest follow-up duration in these studies was only 15 months. Studies with a prolonged follow-up duration are needed to validate this advantage.

The operation time for the advanced minimally invasive technique is generally longer than that for conventional techniques. The average operation time per level of UBE decompression is 64–77 min, which is shorter than the time required for microendoscopic decompression (94–126 min) [46,47,48]. The shorter operation time may be attributable to easy bleeding control and an improved surgical field of view in UBE surgery. The short operation time in UBE surgery is important because the procedure is performed under a continuous flow of normal saline. If the operation is prolonged, the hydrostatic pressure of normal saline may induce elevation of intracranial pressure and resultant neurological complications [30,49]. However, to the best of our knowledge, no study has reported such complications induced by the hydrostatic pressure of normal saline used in UBE surgery.

Intraoperative blood loss was reported in only three studies using the interlaminar approach, one study using the contralateral approach, and none of the studies using the paraspinal approach [6,14,18,50]. A continuous flow of normal saline suppressed the bleeding from small epidural vessels and cancellous bones. The small amount of bleeding was diluted and drained out by the continuous outflow of normal saline. Therefore, such a small amount of blood loss does not cause any major harm to patients.

The complication rate appears to be closely associated with surgeons’ learning curves for this new technique. As mentioned in the earlier text, a dural tear was found to be the most common complication in UBE decompression for DLSS, followed by epidural hematoma. Kim et al. reviewed 1551 consecutive patients who had undergone UBE surgery; the incidence of dural tear was 1.6%, and 52% of the total incidents occurred during the first 6 months of practice [51]. Familiarity with the UBE technique markedly reduced the incidence of this complication. The advanced contralateral approach is generally performed by surgeons with considerable levels of relevant experience. This may explain the lack of articles reporting dural tears in UBE surgery performed using the contralateral approach.

Heo et al. identified retroperitoneal fluid collection to be an unusual complication of the paraspinal approach [33]. The continuous flow of normal saline in UBE surgery creates and maintains a clear surgical field. During foraminal stenosis decompression performed using the paraspinal approach, the boundary of the paraspinal muscles and retroperitoneal space may be damaged without being apparent during surgery. High-pressure normal saline may leak into the retroperitoneal space, leading to retroperitoneal fluid collection and postoperative abdominal pain. Decreasing the hydrostatic pressure of normal saline and avoiding the unnecessary dissection of the paraspinal muscles may prevent this complication.

Several factors limit the generalizability of our findings. First, the studies we reviewed varied in terms of diagnostic criteria, patient selection, and surgeons’ surgical skills. Thus, heterogeneity across the included studies was inevitable. Second, patients with spondylolisthesis or scoliosis were excluded from most studies. Therefore, our findings are not applicable to patients with DLSS associated with preoperative segmental instability. Third, the sample size was small in most of the included studies, particularly in those on the contralateral and paraspinal approaches. Fourth, the follow-up periods were relatively short for most studies, which resulted in a lack of long-term data. Hence, future studies with long-term follow-up are warranted to evaluate delayed outcomes, such as restenosis, post-decompression segmental instability, and reoperation. Finally, most studies included in this review were cohort or case series studies. Prospective comparative studies and randomized controlled trials are needed to compare the advantages and disadvantages of this new technique with those of conventional surgical techniques.

## 5. Conclusions

The interlaminar, contralateral, and paraspinal UBE approaches can be used for various degenerative pathological conditions of the lumbar spine. The clear endoscopic view of the surgical field and ergonomic maneuvering of the surgical instruments enable surgeons to meticulously decompress stenosis to ensure satisfactory surgical outcomes. Nonetheless, prospective long-term studies with large cohorts are warranted to validate the advantages of this new technique.

## Figures and Tables

**Table 1:** Characteristics of articles describing the interlaminar approach.

Study	Year	Study Design	Number of Patients/Levels Operated	Male/Female	Age (Years)	Indication	Lumbar Segment	Follow-Up (Months)
Hua et al. [16]	2022	Retrospective comparative study	36/36	15/21	57.3 ± 10.9	Single-level lumbar spinal canal stenosis	L2-3:1 L3-4:1 L4-5:27 L5-S1:7	12
Ito et al. [17]	2021	Retrospective comparative study	42/42	16/28	66.3 ± 12.3	Single-level lumbar spinal canal stenosis	L3-4:13 L4-5:24 L5-S1:5	6
Aygun et al. [18]	2021	Prospective comparative study	77/77	44/33	64.64 ± 10.9	Single-level lumbar spinal canal stenosis	N/A	24
Park et al. [19]	2020	Randomized controlled trial	32/32	13/19	66.2	Single-level lumbar spinal stenosis	L2-3:2 L3-4:5 L4-5:25	12
Pao et al. [20]	2020	Retrospective case series	81/ 105	38/43	70.2 ± 10.8	Lumbar spinal canal stenosis	T11-12:1 L1-2:1 L2-3:4 L3-4:28 L4-5:67 L5-S1:4	8.6
Min et al. [21]	2020	Retrospective comparative study	54/54	27/27	65.74 ± 10.52	Lumbar central stenosis or lateral recess stenosis without foraminal stenosis	L2-3:1 L3-4:7 L4-5:43 L5-S1:2	27.2 ± 5.4
Kim et al. [22]	2020	Retrospective comparative study	30/30	13/17	64.23 ± 5.26	Lumbar central canal stenosis	L2-3:2 L3-4:8 L4-5:18 L5-S1:2	12
Kim et al. [23]	2019	Retrospective case series	58/58	25/33	63.1 ± 11.8	Severe and focal lumbar spinal canal stenosis	L3-4:10 L4-5:46 L5-S1:2	18
Kang et al. [24]	2019	Randomized controlled trial	32/32	13/19	65.1 ± 8.6	Single-level lumbar spinal canal stenosis	L3-4:4 L4-5:16 L5-S1:12	6
Heo et al. [25]	2019	Retrospective comparative study	37/37	15/22	66.7 ± 9.4	Single-level lumbar central and lateral recess stenosis at L4-L5	L4-5:37	12.5 ± 3.3
Choi et al. [26]	2019	Retrospective comparative study	35/35	14/21	65.4 ± 11.8	Lumbar spinal canal stenosis	N/A	6
Kim et al. [27]	2018	Retrospective case series	105/ (N/A)	46/59	71.2 ± 8.9	Lumbar spinal canal stenosis	N/A	14
Heo et al. [28]	2018	Prospective comparative study	46/ 46	18/28	65.8 ± 8.9	Single-level lumbar central stenosis	L2-3:1 L3-4:8 L4-5:33 L5-S1:4	14.5 ± 2.3
Torudom et al. [29]	2016	Retrospective case series	30/35	11/19	56 ± 6.2	Lumbar spinal stenosis	L4-5:21 Others: N/A	24
Eum et al. [30]	2016	Retrospective case series	58/58	18/40	63.4 ± 7.4	Single-level lumbar spinal stenosis	L3-4:9 L4-5:44 L5-S1:5	13.8 ± 3.3
Soliman [14]	2015	Retrospective case series	94/214	38/56	52	Lumbar spinal stenosis	L2-3:28 L3-4:72 L4-5:90 L5-S1:24	28

N/A, not available.

**Table 2:** Characteristics of the articles describing the contralateral approach.

Study	Year	Study Design	Number of Patients/Levels Operated	Male/Female	Age (Years)	Indication	Lumbar Segment	Follow-Up (Months)
Heo et al. [31]	2019	Retrospective case series	10/10	5/5	57.3 ± 14.7	Lumbar juxtafacet cyst	L3-4:4 L4-5:6	10.1 ± 5.2
Akbary et al. [6]	2018	Retrospective case series	30/30	15/15	61	Degenerative lumbar spinal stenosis at two contiguous levels and unilateral radiculopathy	L2-3:2 L3-4:7 L4-5:12 L5-S1:9	5.67 ± 3.5

**Table 3:** Characteristics of the articles describing the paraspinal approach.

Study	Year	Study Design	Number of Patients/Levels Operated	Male/Female	Age (Years)	Indication	Lumbar Segment	Follow-Up (Months)
Park et al. [32]	2021	Retrospective case series	35/35	16/19	68.4 ± 6.6	Extraforaminal stenosis at L5-S1	L5-S1:35	14.9 ± 4.2
Heo et al. [33]	2019	Retrospective case series	16/16	4/10	59.5 ± 7.2	Unilateral extraforaminal entrapment of the L5 nerve root (far out syndrome)	L5-S1:16	11 ± 5.0
Kim et al. [34]	2018	Retrospective case series	31/31	14/17	70.5 ± 8.9	Lumbar foraminal stenosis	L2-3:3 L3-4:1 L3-4-5:2 L4-5:12 L4-5-S1:2 L5-S1:11	14.8 ± 1.6
Ahn et al. [35]	2018	Retrospective case series	21/27	10/11	64.2 ± 10.7	Lumbar foraminal stenosis	L1-2:1 L2-3:4 L3-4:9 L4-5:6 L5-S1:7	14.8 ± 2.96

**Table 4:** Clinical outcomes indicated in the articles reviewed in the present study.

Study	Approach	Operation Time per Level (min)	Preoperative			Final Follow-Up			Macnab * (%)	Complications
			VAS Leg Pain	VAS Back Pain	ODI	VAS Leg Pain	VAS Back Pain	ODI		
Hua et al. [16]	Interlaminar	69.4 ± 18.5	7.0	5.4	51.4	1.4	1.9	19.8	94.4	Dural injury:2
Ito et al. [17]		57 ± 10.3	3.9	3.9	23.5	1.0	1.3	11.3	N/A	Dural injury: 2
Aygun et al. [18]		57.74	N/A	N/A	53.18	N/A	N/A	8.26	92	N/A
Park et al. [19]		67.2 ± 19.8	6.5	6.1	46.2	2.61	2.75	19.79	N/A	Dura injury: 2 Postoperative hematoma: 1
Pao et al. [20]		89 ± 56.9	7.3	4.3	54.6	0.9	1.2	14.6	93.8	Dura injury: 4 Transient weakness: 1 Epidural hematoma: 1 Inadequate decompression: 1
Min et al. [21]		53.6 ± 6.7	7.38	5.27	60.4	1.48	1.64	15.4	83	Dural injury: 2 Epidural hematoma: 1
Kim et al. [22]		58.1 ± 6.04	N/A	7.13	71.2	N/A	1.23	23.53	76.66	Dural injury: 1
Kim et al. [23]		N/A	7.9	7.1	N/A	1.6	1.9	N/A	93.1	Dural injury: 2
Kang et al. [24]		36 ± 11	N/A	6.3	55	N/A	1.6	5	N/A	Postoperative hematoma: 1
Heo et al. [25]		62.4 ± 5.7	8.05	7.02	58.68	2.16	1.95	23.14	N/A	Dural injury: 1 Postoperative hematoma: 1
Choi et al. [26]		N/A	6.3	6.8	N/A	2.2	2.8	N/A	N/A	Dural injury: 2 Root injury: 1
Kim et al. [27]		53 ± 13.5	7.7	N/A	67.4	2.4	N/A	22.9	88	Dural injury: 2 Postoperative hematoma: 1
Heo et al. [28]		61.1 ± 5.2	7.96	7.04	57.98	2.07	1.98	21.98	N/A	Dural injury: 1 Postoperative hematoma: 1
Torudom et al. [29]		98.3 ± 14.3	8.3	7.2	65.2	2.3	2.4	24	83	Transient paresthesia: 2
Eum et al. [30]		68.9 ± 16.1	8.3	N/A	67.2	2.4	N/A	24.3	81	Postoperative headache: 3 Dural injury: 2 Transient leg numbness: 2 Postoperative hematoma: 1
Soliman [14]		62.8	N/A	N/A	64.2	N/A	N/A	N/A	87	Dural injury: 6
Heo et al. [31]	Contralateral	60.1 ± 23.4	N/A	7.64	45.35	N/A	1.63	15.82	NA	Transient hypoesthesia: 1; Postoperative epidural hematoma: 1
Akbary et al. [6]		102.5 ± 43.66	N/A	N/A	67.9	N/A	N/A	15.7	N/A	0
Park et al. [32]	Paraspinal	63.5 ± 14.4	7.23	3.71	61.5	2.26	2.34	28.6	80	0
Heo et al. [33]		72.8 ± 15.5	8.4	N/A	60.2	2.8	N/A	22.1	71.4	Perirenal fluid collection (abdominal pain): 1
Kim et al. [34]		48.7 ± 13.9	7.87	5.13	66.81	1.45	1.52	17.39	80.6	0
Ahn et al. [35]		96.7 ± 25.9	7.5	N/A	N/A	2.5	N/A	N/A	80.9	Dural injury: 1
Yeung et al. [15]	Interlaminar	69 ± 25.1	Improvement in VAS scores for leg pain: 69.3%						N/A	0
	Contralateral	69.2 ± 35.6	Improvement in VAS scores for leg pain: 63.6%						N/A	Persistent right leg pain: 1

VAS, Visual Analog Scale; ODI, Oswestry Disability Index, N/A, not available, * Ratio of good and excellent results.

**Table 5:** A synthesis of three approaches.

	Interlaminar	Contralateral	Paraspinal
Number of studies	17	3	4
Total number of patients	884	74	103
Mean age (years)	64.11 (range, 52–71.2)	61.37 (range, 57.3–65.8)	65.65 (range, 59.5–70.5)
Most frequently operated level	L4-5 (45.7%)	L4-5 (45%)	L5-S1 (56.9%)
Mean operation time (min)	64.24 (range, 36–98.3)	77.27 (range, 60.1–102.5)	70.43 (range, 48.7–96.7)
Complications	5.7%	4.05%	1.94%
Improvement in VAS scores for leg pain	73.46%	63.6%	70.9%
Improvement in VAS scores for back pain	68.96%	78.66%	53.65%
Improvement in ODI scores	67.41%	71%	67.17%
Macnab (%) *	87.22	N/A	78.23

VAS, Visual Analog Scale; ODI, Oswestry Disability Index, N/A, not available, * Ratio of good and excellent results.

## Data Availability

Data are available upon reasonable request.

## Supplementary Materials

The following supporting information can be downloaded at: https://www.mdpi.com/article/10.3390/diagnostics13061092/s1, File S1: PRISMA checklist for systematic review; File S2: Tables S1–S3.
