# Supplementary material for: Indications for and Outcomes of Three Unilateral Biportal Endoscopic Approaches for the Decompression of Degenerative Lumbar Spinal Stenosis: A Systematic Review
Source: Diagnostics (Basel). 2023 Mar 14;13(6):1092. doi: 10.3390/diagnostics13061092 (PMC10047819; doi:10.3390/diagnostics13061092)
Supplement: Supplementary file 1 [file diagnostics-13-01092-s001.zip › Supplementary material S2.pdf]

Supplementary Table S1. National Institutes of Health (NIH) quality assessment tool for case-control study

**Study**	Q1	Q2	Q3	Q4	Q5	Q6	Q7	Q8	Q9	Q10	Q11	Q12	Quality rating
Hua et al., 2022 [16]	Yes	Yes	NR	NR	Yes	Yes	NA	No	Yes	Yes	NR	Yes	Good
Ito et al., 2021 [17]	Yes	Yes	NR	Yes	Yes	Yes	NA	No	Yes	Yes	NR	NR	Good
Aygun et al., 2021 [18]	Yes	Yes	NR	Yes	Yes	Yes	Yes	No	Yes	Yes	Yes	NR	Good
Min et al., 2020 [21]	Yes	Yes	NR	NR	Yes	Yes	NA	No	Yes	Yes	NR	NR	Fair
Kim et al., 2020 [22]	Yes	Yes	NR	NR	Yes	Yes	NA	No	Yes	Yes	NR	NR	Fair
Heo et al., 2019 [25]	Yes	Yes	NR	NR	Yes	Yes	NA	No	Yes	Yes	NR	NR	Fair
Choi et al., 2019 [26]	Yes	Yes	NR	NR	Yes	Yes	NA	No	Yes	Yes	NR	Yes	Fair
Heo et al., 2018 [28]	Yes	Yes	NR	Yes	Yes	Yes	Yes	No	Yes	Yes	Yes	NR	Good
Yeung et al., 2022 [15]	Yes	Yes	NR	NR	Yes	Yes	NA	No	Yes	Yes	Yes	NR	Fair

Q1: Was the research question or objective in this paper clearly stated and appropriate?; Q2: Was the study population clearly specified and defined?; Q3: Did the authors include a sample size justification?; Q4: Were controls selected or recruited from the same or similar population that gave rise to the cases (including the same timeframe)?; Q5: Were the definitions, inclusion and exclusion criteria, algorithms or processes used to identify or select cases and controls valid, reliable, and implemented consistently across all study participants?; Q6: Were the cases clearly defined and differentiated from controls?; Q7: If less than 100 percent of eligible cases and/or controls were selected for the study, were the cases and/or controls randomly selected from those eligible?; Q8: Was there use of concurrent controls?; Q9: Were the investigators able to confirm that the exposure/risk occurred prior to the development of the condition or event that defined a participant as a case?; Q10: Were the measures of exposure/risk clearly defined, valid, reliable, and implemented consistently (including the same time period) across all study participants?; Q11: Were the assessors of exposure/risk blinded to the case or control status of participants?; Q12: Were key potential confounding variables measured and adjusted statistically in the analyses? If matching was used, did the investigators account for matching during study analysis?. NA = Not Applicable; NR = Not Reported.

Supplementary Table S2. National Institutes of Health (NIH) quality assessment tool for case-series study

**Study**	Q1	Q2	Q3	Q4	Q5	Q6	Q7	Q8	Q9	Quality rating
Pao et al., 2020 [20]	Yes	Yes	Yes	Yes	Yes	Yes	Yes	No	Yes	Good
Kim et al., 2019 [23]	Yes	Yes	Yes	Yes	Yes	Yes	Yes	Yes	Yes	Good
Kim et al., 2018 [27]	Yes	CD	NR	Yes	Yes	Yes	Yes	Yes	Yes	Good
Torudom et al., 2016 [29]	Yes	Yes	Yes	Yes	Yes	Yes	Yes	Yes	Yes	Good
Eum et al., 2016 [30]	Yes	Yes	NR	Yes	Yes	Yes	Yes	Yes	Yes	Good
Soliman et al., 2015 [14]	Yes	Yes	Yes	Yes	Yes	Yes	Yes	Yes	Yes	Good
Heo et al., 2019 [31]	Yes	Yes	Yes	Yes	Yes	Yes	Yes	NA	Yes	Good
Akbary et al., 2018 [6]	Yes	Yes	Yes	Yes	Yes	Yes	CD	Yes	Yes	Good
Park et al., 2021 [32]	Yes	Yes	NR	Yes	Yes	Yes	Yes	Yes	Yes	Good
Heo et al., 2019 [33]	Yes	Yes	Yes	Yes	Yes	Yes	Yes	Yes	Yes	Good
Kim et al., 2018 [34]	Yes	CD	NR	Yes	Yes	Yes	Yes	Yes	Yes	Good
Ahn et al., 2018 [35]	Yes	Yes	Yes	Yes	Yes	Yes	Yes	Yes	Yes	Good

Q1: Was the study question or objective clearly stated?; Q2: Was the study population clearly and fully described, including a case definition?; Q3: Were the cases consecutive?; Q4: Were the subjects comparable?; Q5: Was the intervention clearly described?; Q6: Were the outcome measures clearly defined, valid, reliable, and implemented consistently across all study participants?; Q7: Was the length of follow-up adequate?; Q8: Were the statistical methods well-described?; Q9: Were the results well-described?. CD = Cannot Determine; NA = Not Applicable; NR = Not Reported.

Supplementary Table S3. Risk of Bias assessment using the ROB2.0 for randomized controlled trial study.

Study	Randomization process	Deviations from intended interventions	Missing outcome data	Measurement of the outcome	Selection of the reported results	Overall
Park et al., 2020 [19]	Low risk	Low risk	Low risk	Low risk	Low risk	Low risk
Kang et al., 2019 [24]	Low risk	Some concerns	Low risk	Low risk	Low risk	Some concerns
